# Infancy and Childhood

**Published:** 1913-11

**Authors:** David Allyn Gorton

**Affiliations:** Brooklyn, N. Y.


### Infancy and Childhood.

BY DAVID ALLYN GORTON, M. D., BROOKLYN, N. Y.

The human infant at birth is the most fragile of all vertebrated animals. It is as tender as the tiniest flower; it is often as fragile as a snow-flake. It has the least instinct of all animals of its species, and it only reasons potentially. The young mammals of other vertebrates have instinct equal to their wants and the regulation of their habits. The mother of other vertebrates have instinct equal to every exigency of the puerperal state, foreseeing the time of travail, and making provision for the needs of their young. It is not so with the human mother. She is supposed to have maternal instinct and to know naturally how to care for and rear her young, but it is an error. The human mother oftener has neither instinct nor intuition to guide her for such duties. It is necessary, therefore, that she should be educated in these things. Even mothers experienced in child bearing need scientific instruction to properly care for their infants, not only the first year of its age, but to the age of puberty. The ignorant, experienced mother is often so filled with antiquated and superstitious notions as to unfit her care for her baby. I have known such mothers to give a little fresh urine to the new-born babe. The custom of masticating solid food for the infant of a few months old, by the mother, may still be observed in the back districts. The taut "belly band," and the tightly pinned band of the petticoat are still in vogue in sections less remote from civilization, put on the infant by the motherly "instinct."

Nearly half of the children of Christendom die before reaching the age of five years. It is strange that such a fact should not awaken more widespread public interest. It is a trite saying, that "the good die young." As a matter of fact, it is true as to infancy, but how misleading it is. Death comes to many of these unfortunate waifs of inhumanity, mercifully, no doubt. But it would be just and wise on the part of a Divine Providence to damn a community, or State, that permitted such merciless and inhuman care of our young and defenseless progeny. The chief causes of this shocking mortality of infants, are ignorance and neglect.

### FEEDING OF INFANTS.

The natural food of all young mammals is milk. The milk of the healthy human mother never disagrees with her infant. The nutrition that nature secures the human infant is as perfect in its adaptation to the wants of the baby as that which she provides for the young of the lower mammals. It is true that mother's milk frequently disagrees with her baby, being of poor quality, or other ways hurt by the mal-condition of the mother. The mother not infrequently loses her milk from some abnormal cause, and so-called artificial food must be substituted. In the preparation of such artificial food for infants, the chemists have been at work since the time of Liebig, half a century or more ago. His formula for the preparation of food for infants is still used, with certain modifications, in the form of Mellin's Food.

It is too much to affirm that the chemico-physiologists have ever succeeded in perfectly imitating mothers' milk. Nevertheless, they have succeeded in so modifying cows' milk as to make it digestible by the infant. The chief agency in this process is malt, with finely prepared cereals, wheat, barley, the albumen of eggs, etc., which is found to soften and otherwise change the casein of cows' milk so as to render it easily digestible in the stomach of the human infant.

These observations are intended to apply to the first six months of a child's existence, when it is presumed to have cut its first four incisors. It is hardly necessary to say, that no other food whatever, water excepted, is admissible for the infant at this period, and long after it in most cases. In this period the infant is most delicate, and needs to be guarded with greatest care against errors in feeding. The custom of giving the baby a taste of a little fruit jelly, a chicken bone to pick, or a rind of bacon to suck, is reprehensible. No mother with a proper regard for the comfort and well-being of her child will indulge a custom so pernicious.

As dentition goes forward and other teeth come, and the irritation of teething subsides, certain other simple things, as Graham and oat-meal crackers, the juicy fruits, as the apple, banana, pear, peach, the juice of sweet grapes, bread-and-butter, etc., will have their place in the child's dietary. The acid fruits should be avoided, and every form of animal food, except, of course, milk, which after seven months, may be given unmodified, in most instances. Crackers made of fine bolted wheat flour have no place in a child's diet.

It should be borne in mind that the stage of dentition is the natural guide to the infant's dietary. While I am persuaded that man is an omnivorous animal by nature, I am equally persuaded that a frugivorous diet is best for him, that is, to perfect a sound mind in a sound body. If one would develop selfish, combative children, give them flesh food and give it early, or at the end of their second year. They will be likely to have their first complement of teeth at that age, and be able to masticate, and should have food to masticate. The fruits and the cereals, preferably, as I have stated take rank. Guard against excess of the carbon foods. It is an error to suppose that a fat child is properly nourished. The advertising booklets of food for infants give illustrations of children, rosy, fat, and beautiful. The illustrations may be true to life, but they are misleading in fact. In the nutrition of an infant, the aim should be harmonious development, of body and mind, and the latter should be the leading consideration in the infant's second year, I mean as to nourishment, not of training. There are articles of food that minister more especially to mentality, others to the bony structure, still others to the cellular tissue, others to intellect. The last should be kept in mind in selecting an infant's dietary.

### HYGIENE OF THE NURSERY.

The nursery is the baby's little world. Let it be all its own, and constitute an environment for the newly born of health and comfort. The apartment should be spacious, with ample means of ventilation and sunshine. Its floor should be bare of carpets and rugs. Let there be no draperies to gather and conceal death-dealing dust. The floor is the baby's playground. It should be kept clean and sweet. No dust, soiled napkins, or unclean clothing, should be allowed to accumulate. In this department place a couch, thoroughly protected from dampness, where the baby can sleep, away from the noise of the street or the household. On its door let it be writ, "No smoking allowed here."

### NURSING APHORISMS.

1. Be in no hurry to bathe the new-born infant. A few hours of rest after a tedious labor is as necessary for it as for the mother.

2. Unless there are special reasons for a belly-band, it should be omitted.

3. The baby should be put to the breast as soon as the mother is prepared to receive it.

4. Be in no hurry to feed the baby. It is not ready for food until the mother is prepared to furnish it. Meantime, give the child from time to time a little warm water slightly sweetened with honey.

5. If the mother's milk fails, and artificial food be substituted, look well after the nursing bottle and nipple. The nursing bottles should be aseptically cleansed with boiling water before each using; the nipple also, especially in warm weather.

6. The baby should be bathed daily in warm soft water, to which a modicum of soap has been added. As it increases in strength, the bath should be more frequent and cold. Wet napkins should be removed promptly, and the child kept dry and sweet.

7. The new-born infant is often as delicate as a flower; handle it carefully and as little as possible.

8. Be patient with the crying baby. It is unhappy or suffering, and may be ill.

9. No healthy baby will cry, except from hunger or thirst; the need of being changed, or some concealed want.

10. Beware how you allow a baby to sleep in soiled or wet diapers to absorb urea, which is almost as poisonous as nicotine.

11. Be regular as to intervals of feeding. The healthy baby, during its first few months, should be fed every three hours, from early morning until midnight. The weakling may need to be fed less at a time and every two hours, and sometimes oftener.

12. Never give the baby its food cold. Confine the child to one article of food, until a change of diet is necessary. Give the baby as much food at one time as it will take; there is no danger from over-feeding; the child will promptly return any surplus it may have taken.

13. Should an infant seem to require food between the regular intervals of feeding, it is probably thirsty rather than hungry; give it warm water (sterilized) made palatable with sugar or honey.

14. No healthy baby after the age of seven months, needs to be fed oftener than four-hour intervals, and should not be fed at all between the hours of 7 a. m. and 11 p. m.

15. If the infant be brought up on the bottle, the problem is one of nutrition. No child could become seriously ill, barring accidents and contagious diseases, that is well nourished; the nourished child digests its food.

16. Watch the dejections of the child that is brought up on modified milk. You will find in them how and when to change the proportion of the modifying element to the proportion of the milk.

17. The milk of the baby should be as fresh as possible. Milk is extremely sensitive to morbific causes and even harmless odors and gases and may be spoiled for the baby's use in the ice-box, from odors that are sweet and harmless in themselves. A foul ice-box is, therefore, not the only source of infection of milk.

18. The colicky infant may usually be relieved by the use of water as hot as can be prudently given, made palatable with honey, and administered by the nursing bottle. Relief by this means is to be preferred to the ministration of the customary soothing medicines. No child will have colic that digests its food.

19. Do not give an infant either laxative or astringent medicines. Nor should the baby be given anoynes of any kind, or for any purpose. There are wiser and more rational methods of meeting the indications for which they are prescribed. A drop of laudanum has been known to kill a young infant.

20. Let no mother worry over dentition, nor fear the dangers of the "second summer." Look well to the digestion and nutrition of the baby; keep the alimentary tract unobstructed and free of ptomaines and other crudities, and there will be no danger of choleras, dysentaries and convulsions. The "second summer" would then be an improvement over the first.

21. Do not encourage precocity. Leave the infant, the first few months, almost entirely to nature, that is, to itself. The brainy child has everything to lose by over-attention; the brainless child, nothing to gain.

22. Dress the baby lightly in wool of soft light texture in cold weather; in warm weather it should be of linen or silk. Do not put on stockings and shoes nor afflict it with caps and bands and long swaddling clothes.

23. Never load the baby down with wraps, or cover it at any time with heavy, impervious blankets, or quilts. An hour's sweating will neutralize a week's growth. Put the baby to bed in fresh, clean garments.

24. Remember that the skin is a breathing organ, especially an infant's skin. During the first few weeks of its life, it breathes more by its skin than by its lungs. Do not asphyxiate the child with wealth of wraps nor poison it to death with retained wet diapers.

25. Constipation is the infant's peril. If the child be constipated, and its stools dry, and difficult to pass, or too large to pass easily, the warm water rectal douche should be given freely but judiciously. Should this procedure not afford prompt relief to the little sufferer, use sweet oil instead of water. Laxative medicine is too slow to act in such an emergency, and if given, would afford only temporary relief, and leave the child in worse condition than it was at first. The radical cure of such a condition should be sought in the baby's dietary.

In attaching so much importance to hygiene and dietetics in the care and treatment of infants and children, let no one conclude that we underrate the importance of medicaments in the treatment of certain abnormal conditions to which the infant, through bad heredity and a worse environment, falls a victim. Medicaments have a wide range of usefulness in pediatrics, of course, but it was foreign to our purpose to intrench upon that branch of our subject, since we have not written so much for the profession as for nurses and mothers, with the object of extending or enlarging the perspectives of their duties and responsibilities in the family and home. Hygiene and sanitation are complements of medicine, the science of which is entirely due to the unselfish labors of the medical profession.—*Med. Times,* October, 1913.

---

There's a bird in the Zoo named a Pelican,
Whose mouth holds more than his Belican,
He can keep in his beak
Enough food for a week,
But I don't see how in the Helican!—*Life.*

